# Perks of Rehabilitation in Improving Motor Function in a Nine-Year-Old Male With Duchenne Muscular Dystrophy: A Case Report

**DOI:** 10.7759/cureus.30162

**Published:** 2022-10-11

**Authors:** Purva S Shahade, Purva H Mundada, Snehal S Samal

**Affiliations:** 1 Physiotherapy, Ravi Nair Physiotherapy College, Datta Meghe Institute of Medical Sciences, Wardha, IND; 2 Kinesiology, Ravi Nair Physiotherapy College, Datta Meghe Institute of Medical Sciences, Wardha, IND

**Keywords:** rehabilitation, range of motion, hypotonia, gower’s sign, muscle weakness, duchenne muscular dystrophy

## Abstract

Duchenne muscular dystrophy (DMD) is a progressive disorder. It is the most typical X-linked muscular dystrophy in children. It primarily affects males and is characterized by motor delays, muscle weakness, respiratory impairment, and loss of ambulation. A case study of a nine-year-old male diagnosed with DMD is presented. The patient had difficulty walking since the age of four and had a proximal lower extremity weakness on both sides previously with a reduced range of motion (ROM). For the last 15 days, the patient is unable to walk and has hypotonia in both lower limbs. The diagnostic Gowers sign was positive. Balance and mobility were affected. Treatment of this patient is focused mostly on maintaining the range of motion (ROM), respiratory training, and balance.

## Introduction

Duchenne muscular dystrophy (DMD) is one of the most severe types of muscular dystrophy [1]. It is an X-linked recessive muscular dystrophy [2]. It is caused by a mutation in the dystrophin gene, which leads to the absence or decrease in dystrophin [1]. DMD is one of the progressive neuromuscular diseases [3]. It is a genetic condition [4]. It primarily affects boys [5].

It is characterized by motor delays, muscle weakness, respiratory impairment, and loss of ambulation [6]. Muscle weakness in DMD affects the proximal muscles more than the distal muscles and hence begins in the lower limbs first [7]. The progression of this disease consists of five stages: presymptomatic stage, early ambulatory stage, late ambulatory stage, early non-ambulatory stage, and late non-ambulatory stage [2]. Although it is a genetic disorder, it may happen that individuals who do not even have a family history of DMD also get the disease when their genes are affected on their own [7]. Diagnosis usually occurs at approximately age five. Symptoms include Gowers sign, frequent falls, and trouble running and climbing stairs [1,2]. More than 75% of DMD cases are usually inherited from the mother, whereas approximately 25% of cases are due to a mutation in the gene for dystrophin [1]. People with DMD do not produce dystrophin at all or produce a small amount of it in their muscles, and due to the absence of dystrophin, even everyday activity can cause huge damage to the muscle cells [8]. A case study of a nine-year-old male diagnosed with DMD is presented here. This study included physical therapy assessment and intervention strategies.

## Case presentation

Patient information

A nine-year-old male patient who was a resident of Amravati district and a second-class student was diagnosed with DMD at around the age of four when his father consulted a doctor at Amravati after noticing his difficulty in walking and weakness. He was then referred to Acharya Vinoba Bhave Rural Hospital (AVBRH), Wardha, for further treatment. He came to AVBRH on January 21, 2021. The patient was apparently alright until the age of four, after which he began to walk on his toes, which has been later on followed by an inability to walk for 15 days before he consulted AVBRH. His inability to walk was also associated with vomiting, which was non-projectile and non-bilious, with non-blood tinged in small quantities, and containing food particles. There was also a history of fever that lasted for three days before consulting at AVBRH. He had hypotonia in both lower limbs and had been unable to walk for the past 15 days. The patient was mildly malnourished. It was a depressing condition for the family members of the patient as they have low socioeconomic status.

Clinical findings

Significant physical examination and important clinical findings are given in Table 1.

**Table 1:** Significant physical examination and important clinical findings.

Examination	Findings
Cardiovascular system	S1 and S2 present, no murmur
Central nervous system	Conscious and active
Respiratory system	Air entry bilaterally equal, bilaterally clear
Musculoskeletal system	Equinus deformity in both feet, and optimal evaluation of Gowers sign was not possible since the patient was unable to stand

Muscle tone and girth are given in Table 2 and Table 3, respectively.

**Table 2:** Muscle tone of both upper and lower limbs.

Limbs	Right	Left
Upper limb	Normal	Normal
Lower limb	Hypotonia	Hypotonia

**Table 3:** Muscle girth.

Area	Right	Left
Upper arm	15 cm	15 cm
Thigh	25 cm	24 cm
Lower limb	19 cm	19 cm

Reflex and manual muscle testing and range of motion (ROM) findings are given in Table 4 and Table 5, respectively.

**Table 4:** Reflex findings of both upper and lower limbs.

Upper limb	Right	Left
Biceps jerk	2+	2+
Triceps jerk	2+	2+
Lower limb	Right	Left
Knee jerk	1+	1+
Ankle jerk	1+	1+
Babinski sign	Not elicited	Not elicited

**Table 5:** Manual muscle testing and range of motion of both upper and lower limbs. ROM: range of motion

Joint movement	Grade		ROM	
	Right	Left	Right	Left
Shoulder flexion	3	3	180°	180°
Shoulder extension	3	3	38°	35°
Shoulder abduction	2	3	178°	175°
Elbow flexion	3	3	144°	145°
Elbow extension	3	3	0°	0°
Hip flexion	1	1	0°	0°
Hip extension	1	1	0°	0°
Hip abduction	1	1	0°	0°
Hip adduction	1	1	0°	0°
Knee flexion	1	1	0°	0°
Knee extension	1	1	0°	0°
Ankle dorsiflexion	1	1	0°	0°
Ankle plantarflexion	1	1	0°	0°

Postural findings are given in Table 6.

**Table 6:** Posture in sitting and standing.

Posture	
Standing	Cannot be analyzed as the patient is unable to stand for the last 15 days; the patient is not able to sit without handheld support
Sitting	Sits with thoracic kyphosis

**Figure 1:**
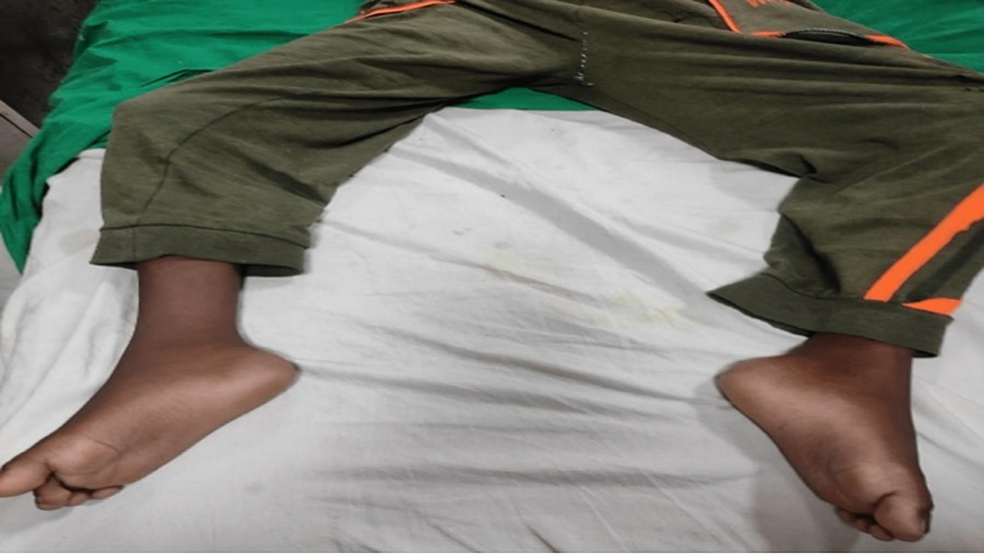
Both lower limbs externally rotated with ankles plantarflexed.

Figure 1 shows the patient’s both lower limbs externally rotated.

Prognosis

DMD is a progressive disorder, and there is no cure for it. Usually, a patient with DMD dies of respiratory infections or cardiomyopathy, but survival can be improved with advances in respiratory care and cardiac care. Steroids can be used to slow the loss of muscle strength.

Medications

Medications are given in Table 7.

**Table 7:** Medications with dosage. Tab: tablet, BD: twice daily, OD: once daily, mg: milligram

Medications	Dosage
Tab Limcee	1 Tab BD
Tab Multivitamin	1 Tab BD
Tab Neurobion Forte	1 Tab OD
Tab Emset	4 mg -½ Tab BD

Corticosteroid therapy

Prednisone and deflazacort are used for the treatment of DMD.

Physiotherapy management

In the physiotherapy management of DMD, a home exercise program can be designed for the patient, and exercises can be given to the patient to slow down the progression of the disease. In DMD, the most important complication a physiotherapist must deal with is contractures in the musculoskeletal system and weakness of respiratory muscles.

Respiratory Training

In DMD, the muscles that assist in breathing get weaker. The patient has been advised to undergo inspiratory muscle training as well as deep diaphragmatic breathing techniques. Respiratory muscle weakness leads to a highly increased risk of lung infection and functional decline [9]. Respiratory training decreases respiratory complications [10,11].

Stretching

Stretching is given to prevent and reduce contractures, and it should be done a minimum of 4-6 days per week [12]. During the ambulatory phase, stretching of the lower extremities is important [13], and when the non-ambulatory phase is reached, stretching the upper extremities is important [12]. Stretching was given for ankle dorsiflexors, knee flexors and extensors, and hip flexors and extensors. Proprioceptive neuromuscular facilitation (PNF) stretching was also given.

Posture

Physiotherapists should observe and analyze a child’s posture in sitting, lying, and standing positions [14]. They must teach the patient or the caregiver how to make the patient lie, sit, stand, and be in appropriate positions using pillows or splints in the most effective ways [15].

Orthotics

Physiotherapists can advise orthosis such as ankle foot orthosis [15].

## Discussion

Duchenne muscular dystrophy is a progressive disorder [1]. It is an X-linked recessive muscular dystrophy [2]. It affects males mostly, and females are usually unaffected, but they can be the carriers [1]. Proximal muscles are affected more than distal muscles, so weakness occurs first in the lower limb and then progresses to the upper limb [7]. Some of the main symptoms the patient may present are delay in receptive and expressive language development and inability to initiate and maintain social contact [16]. Strength training can be beneficial for improving muscular strength and slowing the deterioration of muscle power [17]. The balance and mobility of patients with DMD can be improved by proprioceptive neuromuscular facilitation [18]. Yoga is also beneficial as it is a psychosomatic-spiritual discipline that includes breathing exercises, physical poses, and meditation [19]. As DMD is a depressing condition for the patient and his family, they can be aided with psychotherapy and medicine. Talking therapy can also be used, known as short-term psychotherapy [20].

Birnkrant et al., for the DMD Care Considerations Working Group, presented a case study on DMD diagnosis and management. Their study concluded with guidance on diagnosis and neuromuscular, rehabilitation, gastrointestinal, and endocrine management [1]. Rivera et al. presented a case study on how muscle weakness can be managed medically in DMD cases, and their study concluded that among all corticosteroid regimens present, either deflazacort or prednisone weekend dosing was preferable [3]. Salmaninejad et al. presented a case study on DMD, and their study concluded with updates on the recent gene therapies available for DMD that compensate for the deficiency of dystrophin [4].

The case study of a nine-year-old male with DMD is presented here. The patient had already reached the non-ambulatory stage of DMD. This case study includes the assessment and intervention strategies that are useful to the patient. The assessment consists of body functions and structural impairments assessed through proper assessment of the respiratory system, cardiovascular system, central nervous system, muscle power, muscle bulk, neurological examination, muscle tone, reflex testing, posture and balance assessment, range of motion (ROM), and manual muscle testing. Therapeutic interventions include medications, corticosteroid therapy, and physiotherapy management. Physiotherapy management consists of a home exercise program, respiratory training (breathing exercises), and stretching exercises particularly to reduce contractures.

## Conclusions

This is a classic case of Duchenne muscular dystrophy. It is one of the most severe types of muscular dystrophy in which the patient has already reached the non-ambulatory phase of the disease. This is a progressive disease, so it cannot be reversed, but the progression can be slowed down through effective therapeutic management and proper exercises. An effective home exercise protocol has been designed for the patient, and exercises can be given to the patient to slow down the progression of the disease that also includes respiratory training, orthotics, and stretching. Yoga is beneficial too, as it is a psychosomatic-spiritual discipline that includes a combination of breathing exercises, physical poses, and meditation. Although it is a depressing condition for the patient and the family members and sadly cannot be cured, surely with proper physiotherapy management, the quality of life can be improved.
